# Division of labor: OEX1 and POL1A/POL1B are partial derivatives of prokaryotic DNA polymerase required for mitochondrial genome maintenance in plants

**DOI:** 10.1093/plcell/koaf128

**Published:** 2025-05-22

**Authors:** Renuka Kolli

**Affiliations:** Assistant Features Editor, The Plant Cell, American Society of Plant Biologists; Sainsbury Laboratory, University of Cambridge, Cambridge, UK

Mitochondrial DNA (mtDNA) encodes for essential subunits of oxidative phosphorylation complexes. Maintaining mtDNA integrity is crucial for mitochondrial biogenesis, cell survival, and normal plant growth and development. DNA replication and transcription are 2 essential processes that paradoxically also threaten genome integrity. During replication, lagging strand synthesis is discontinuous, with multiple short stretches called Okazaki fragments, each preceded by an RNA primer. In the Okazaki fragment maturation process, DNA polymerase replaces the RNA primers with DNA nucleotides, and the Okazaki fragments are ligated by DNA ligase to produce an intact lagging strand. The primer removal process can involve strand displacement when DNA polymerase extends an upstream Okazaki fragment into the downstream Okazaki fragment to generate a 5′-flap structure that is then processed by a flap-endonuclease (Zheng and Shen 2011). During transcription, the nascent RNA strand could invade the DNA duplex and get hybridized to 1 of the DNA strands to form a structure known as R-loop, which can affect replication, gene expression, and DNA repair processes (Aguilera and Aguilera 2025). Homologous recombination not only contributes to the highly dynamic structure and rapid evolution of plant mtDNA but also aids in DNA repair (Gualberto and Newton 2017).

Nucleases play a central role in Okazaki fragment maturation, R-loop dissolution, and DNA repair processes. Bacterial Pol I contains an intrinsic 5′-3′-exonuclease/flap-endonuclease domain, which supports replication, DNA repair, and recombination (Figure). Two evolutionarily related plant DNA polymerases, POL1A and POL1B, are dually targeted to mitochondria and plastids (Carrie et al. 2009). However, they lack the 5′-3′-exonuclease/flap-endonuclease domain (Figure). Now, Déborah Schatz and coauthors (Schatz et al. 2025) have identified that Arabidopsis OEX1 (organellar exonuclease 1) compensates for the missing 5′-3′-exonuclease/flap-endonuclease activity of the POL1A/POL1B in the mitochondria (Figure). Moreover, OEX2 was found to be targeted to chloroplasts and likely replaces the missing domain of POL1A/POL1B present there.

**Figure.**
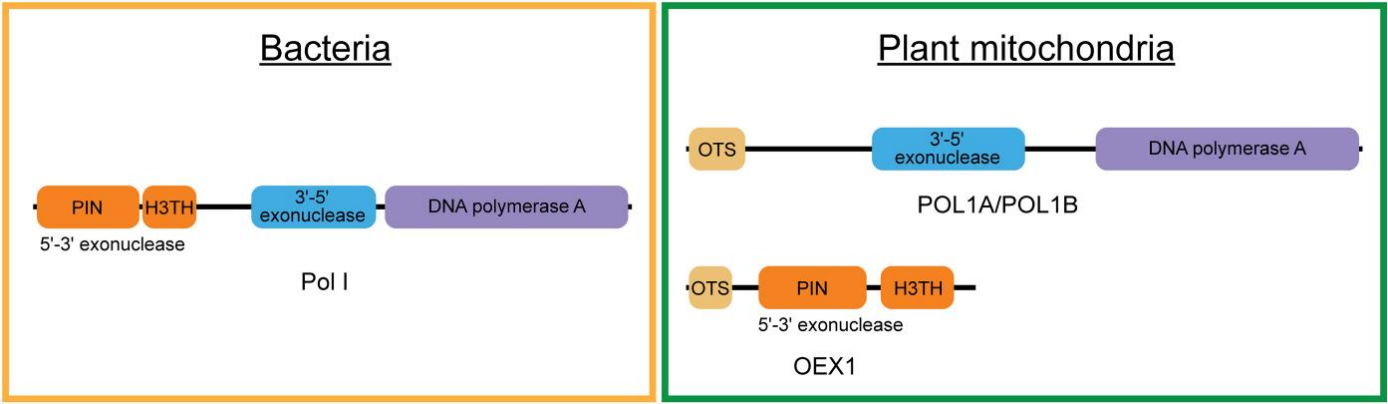
Plant mitochondrial DNA polymerases POL1A and POL1B lack the 5′-3′-exonuclease domain found in bacterial DNA polymerase I. Instead, OEX1 performs the function of this domain. OTS: organellar targeting sequence, PIN: PIN-like nuclease domain, H3TH: helix-3-turn-helix DNA-binding domain. Adapted from Schatz et al. (2025), Figure 1.

In this study, Schatz et al. focused on studying the mitochondrial OEX1 in detail. OEX1 is phylogenetically related to the 5′-3′-exonuclease domain of prokaryotic Pol I and is conserved in the plant lineage. Knockout of OEX1 (*oex1-1*) resulted in severe plant growth and developmental defects that correlated with mtDNA instability as measured by altered stoichiometry and relative copy numbers of different mtDNA regions, particularly in the regions flanked by repeats, indicating altered recombination events. The regions with lower copy numbers did not contain any known genes, and there was no significant decrease in mitochondrial transcript abundances, implicating that the growth defect of *oex1-1* was not due to inadequate expression of mitochondrial genes.

To investigate OEX1 function, the authors conducted in vitro nuclease assays using recombinant OEX1 and a noncatalytic mutated version of the protein to serve as a negative control. The results indicate that OEX1 has 5′-3′-exonuclease and flap-endonuclease activities on double-stranded DNA. The flap-endonuclease activity is especially important for processing Okazaki fragments. OEX1 could also be involved in base excision repair as it extended single-strand gaps on DNA in combination with a DNA polymerase. Additionally, OEX1 was found to have a higher processivity on RNA:DNA hybrids and rapidly degraded the RNA in structures resembling Okazaki fragments and R-loops. Accordingly, the *oex1-1* mutant plants accumulated R-loops in highly transcribed mtDNA regions as evidenced by qPCR following DNA:RNA immunoprecipitation. Therefore, the authors consider mtDNA instability, including problems in replication and differential segregation of recombination-generated mtDNA subgenomes, to have likely resulted in the developmental defects of the mutant plants.

Thus, Schatz et al. (2025) have identified and characterized Arabidopsis OEX1 as a nuclease that functions similarly to the 5′-3′-exonuclease/flap-endonuclease domain of the bacterial DNA polymerase, which is lacking in the plant mitochondrial DNA polymerases (Figure). OEX1 is crucial for mtDNA stability and impacts plant growth and development. Moreover, 2 isoforms of OEX1 were found to be generated by alternative splicing and were named OEX1a and OEX1b. They have similar substrate specificities, but OEX1a displayed relatively higher processivity in vitro on the substrates tested. They appear to have nonredundant roles in maintaining mtDNA stability, which can be investigated in future studies.

## Recent related articles in *The Plant Cell*

- Sloan et al. (2024) reviewed the origins and functions of plant-specific MutS proteins, which affect plant organellar genetics.
- Field et al. (2023) explained, as part of a review, how certain biocondensates that compartmentalize 3′ processing factors can help resolve an R-loop.
- Li et al. (2023) determined that plant apurinic/apyrimidinic endonucleases play a key role in DNA repair in both somatic and meiotic cells.

## Dive Curated Terms

The following phenotypic, genotypic, and functional terms are of significance to the work described in this paper:

OEX2 Gramene: At1g34380
OEX2 Araport: At1g34380

OEX1 Gramene: At3g52050
OEX1 Araport: At3g52050

POL1A Gramene: At1g50840
POL1A Araport: At1g50840

POL1B Gramene: At3g20540
POL1B Araport: At3g20540
